# Association of the *Interleukin-10-592C/A* Polymorphism and Cervical Cancer Risk: A Meta-Analysis

**DOI:** 10.1155/2022/2319161

**Published:** 2022-07-12

**Authors:** Brehima Diakite, Yaya Kassogue, Mamoudou Maiga, Guimogo Dolo, Oumar Kassogue, Jonah Musa, Imran Morhason-Bello, Ban Traore, Cheick Bougadari Traore, Bakarou Kamate, Aissata Coulibaly, Sekou Bah, Sellama Nadifi, Robert Murphy, Jane L. Holl, Lifang Hou

**Affiliations:** ^1^Centre de Recherche et de Formation sur les Pathologies Moleculaires (CREFPAM), University of Sciences, Techniques and Technologies of Bamako (USTTB), Bamako, Mali; ^2^Preventive Medicine Department, Cancer Epidemiology and Prevention, Northwestern University, Chicago, Illinois 60611, USA; ^3^Institute for Global Health, Northwestern University, Chicago, Illinois 60611, USA; ^4^Department of Obstetrics and Gynecology, Faculty of Medical Sciences, University of Jos, Plateau State, Jos, Nigeria; ^5^Department of Obstetrics and Gynecology, Faculty of Clinical Sciences and Institute for Advanced Medical Research and Training (IAMRAT), College of Medicine, University of Ibadan, Ibadan, Oyo, Nigeria; ^6^Faculty of Sciences and Techniques, USTTB, Bamako, Mali; ^7^Faculty of Pharmacy, USTTB, Bamako, Mali; ^8^Hassan II University Aïn Chock, Casablanca, Morocco; ^9^Department of Neurology, University of Chicago, Chicago, Illinois 60611, USA

## Abstract

A literature review showed some discrepancies regarding the association of *-592C/A* with the risk of cervical cancer. To allow more precise analysis of the data by increasing the number of cases studied and more acceptable generalization by considering results from different sources, the present meta-analysis was performed on available published studies that explored the relationship between SNP*-592C/A* of the *IL-10* gene and the risk of cervical cancer. Eleven available studies, including 4187 cases and 3311 controls, were included in this study investigating the relationship between the *-592C/A* polymorphism of *IL-10* and cervical cancer risk. Fixed-effects or random-effects models were performed with pooled odds ratios (ORs). Heterogeneity and bias tests were performed by the inconsistency test and funnel plot, respectively. The overall analysis showed an increased susceptibility to cervical cancer with the *-592C/A* polymorphism of the *IL-10* gene for the recessive model (OR = 1.30, 95% CI = 1.14–1.49), dominant model (OR = 1.36, 95% CI = 1.09–1.70), and additive model (OR = 1.25, 95% CI = 1.09–1.44). Regarding ethnicity, a significant association of the *-592C/A* polymorphism of the *IL-10* gene was linked to an elevated risk of cervical cancer for all genetic models (recessive, dominant, and additive) in the Asian populations and for the recessive and additive models in Caucasians with *P* < 0.05. The *-592C/A* polymorphism of the *IL-10* gene may be considered a risk factor for cervical cancer.

## 1. Introduction

Cervical cancer is the fourth most common cancer in the world, accounting for 6.5% of all cancers, after breast, colorectal, and lung cancer. It is also the fourth highest cause of cancer death in women in both high-income and low-middle-income countries, with an estimated mortality rate of 7.7% worldwide (GLOBOCAN 2020) [1]. Exposure to high-risk human papillomavirus (HPV) is required but is not a sufficient cause of cervical cancer [2, 3]. Oncogenic HPV DNA is present in nearly 100% of invasive cervical cancers. A limited immune response to HPV linked to the host's genetic make-up may increase the risk of cervical cancer [4]. Many authors have been interested in the research of risk factors related to cervical cancer throughout the last few decades [5]. It appears from their investigations that genetic factors seem to exert a significant influence on the carcinogenesis of the cervix. Notably, the reported results are conflicting. However, mutations in genes involved in cytokine synthesis, such as interleukin 10 (IL-10), appear to be strong predictors of cervical cancer risk [6–8]. Physiologically, IL-10 is an essential cytokine for inflammatory modulation. Several cell types are involved in the production of this cytokine, including Th1, Th2, Th17 lymphocytes, B lymphocytes, mast cells, eosinophils, monocytes, macrophages, and dendritic cells [9, 10]. The *IL-10* gene, which contains five exons, has been found on the long arm of chromosome *1q31-32* in humans [11, 12]. The significance of the *IL-10* gene in the control of immune-mediated illness responses has resulted in the discovery of numerous polymorphisms in different portions of the gene, including the promoter region [4, 11]. The majority of identified genetic polymorphisms are single nucleotide polymorphisms (SNPs). The SNP*-592C/A* of the *IL-10* gene promoter is located near a number of transcription factor binding sites. This SNP has been implicated in the pathogenesis of cutaneous malignant melanoma and prostate, breast, gastric, and cervical cancer [6, 13–18]. Association studies carried out in different populations have shown that this SNP increases the risk of developing cervical cancer [6, 17, 19]. However, other studies have reported conflicting results [7, 20, 21]. Furthermore, the expression of the *IL-10* gene and/or the production of IL-10 has been demonstrated in various types of tumors, suggesting that IL-10 could, by promoting escape from the immune system, constitute a step in tumorigenesis [22–24]. Given the contradictory results of numerous studies on the role of SNP*-592C/A* of the *IL-10* gene in the pathogenesis of cervical cancer, we conducted a meta-analysis to assess the association between SNP*-592C/A* in the *IL-10* gene and the risk of cervical cancer.

## 2. Materials and Methods

### 2.1. Literature Search Strategy

The identification of initial manuscripts or published articles, available in English, was performed using the online databases PubMed, the Harvard University Library, Web of Science, and Genetics Medical Literature Database. Additional articles were identified from references cited in relevant reports and journals. The key search words “Interleukin-10” or “IL-10,” “*−592C/A*” or “*−592C* > *A*” or “*rs1800872*,” “polymorphism” or “variant” or “mutation” or “gene” or “cervical tumor” or “cervical cancer” were used to locate and select the articles.

### 2.2. Inclusion Criteria

The eligibility criteria were as follows: (1) case-control study design evaluating the association of the *-592C/A* polymorphisms of the *IL-10* gene with the risk of cervical cancer, (2) availability of the full scientific manuscript, (3) distribution of polymorphisms in the controls in agreement with Hardy–Weinberg equilibrium (HWE), (4) availability of proportions of the different genotypes (*CC*, *CA*, *AA* for *−592C/A* of the *IL-10* gene) in both cases and controls, and (5) no significant change in the value of the odds ratio (OR).

### 2.3. Data Extraction

Three authors independently carried out the literature search to optimize the convergence of the retrieved data, including principal author, year of publication, study design, study population, racial and ethnic groups, sample size, genotypic and allelic proportions in cases and controls, HWE calculation, and genetic models tested.

### 2.4. Statistical Analysis

The statistical analyses were conducted using Review Manager *v5.3* and *MedCalc v14.8.1*. The distribution of the *−592C/A* polymorphism of the *IL-10* gene in agreement with HWE in the controls was evaluated by a chi-square test, with *P* < 0.05. A pooled OR test with a 95% confidence interval (CI) was used to assess the strength of the association between the −*592C/A* polymorphism in the *IL-10* gene and the risk of cervical cancer, including the recessive model (*AA* vs. CC + *CA*), the dominant model (*AA* + *CA* vs. *CC*), and the additive model (*A* vs. *C*). To avoid type I error, the level of significance was corrected using Bonferroni's adjustment during multiple comparisons. An inconsistency (*I*^2^) statistical test was used to determine heterogeneity [25, 26]. If there was no heterogeneity (*I*^2^ < 50%), a fixed-effect model (FEM) was retained for interpretation of a global OR. In the case of heterogeneity, the OR was interpreted using a random-effect model (REM). A funnel plot was used to determine bias [27]. The trial sequential analysis (TSA) software was used to estimate the sample size required for each arm to assess the robustness of the meta-analysis findings with 90% statistical power [28].

## 3. Results

### 3.1. Characteristics of Eligible Studies

**Figure 1:**
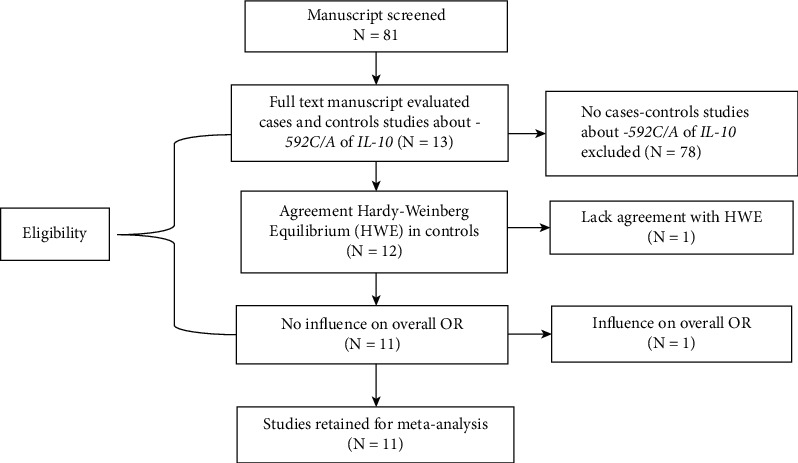
Flow diagram of eligible studies included.

Four Caucasian studies with 2221 cases and 1240 controls [17, 21, 29, 30] and seven Asian studies with 1966 cases and 2071 controls [6, 7, 20, 31–34] were eligible to conduct the current meta-analysis out of thirteen studies (Figure 1) (Table 1). Only one study on Africans was found, and it was discarded due to Hardy–Weinberg's imbalance [8]. Another study that had a major impact on the overall OR was also excluded [19].

### 3.2. Quantitative Analysis

**Figure 2:**
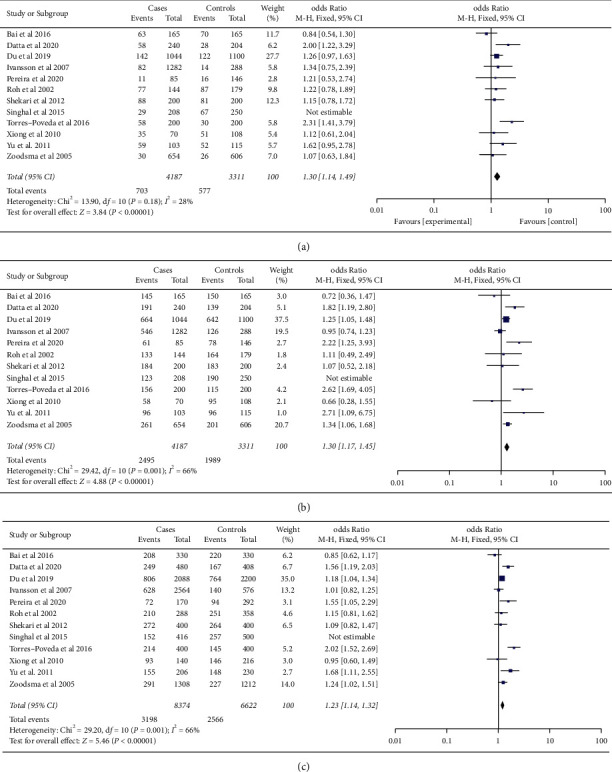
Forest plots of the association between the *-592C/A* polymorphism of the *IL-10* gene and cervical cancer for the (a) recessive model, (b) dominant model, and (c) additive model. The pooled OR is represented by a black diamond, the OR in each study is represented by blue squares with square sizes inversely proportionate to the standard error of the OR, and the horizontal lines represent the 95% CI.

Table 2 denotes the association between cervical cancer and SNP-592C/A of IL-10 for the genetic models. Overall, a significant association was found between the risk of cervical cancer and the three genetic models, including the recessive (OR (FEM) = 1.30, 95% CI = 1.14–1.49, *P*=0.0001), dominant (OR (REM) = 1.36, 95% CI = 1.09–1.70, *P*=0.006), and additive models (OR (REM) = 1.25, 95% CI = 1.09–1.44, *P*=0.001) (Figure 2).

**Figure 3:**
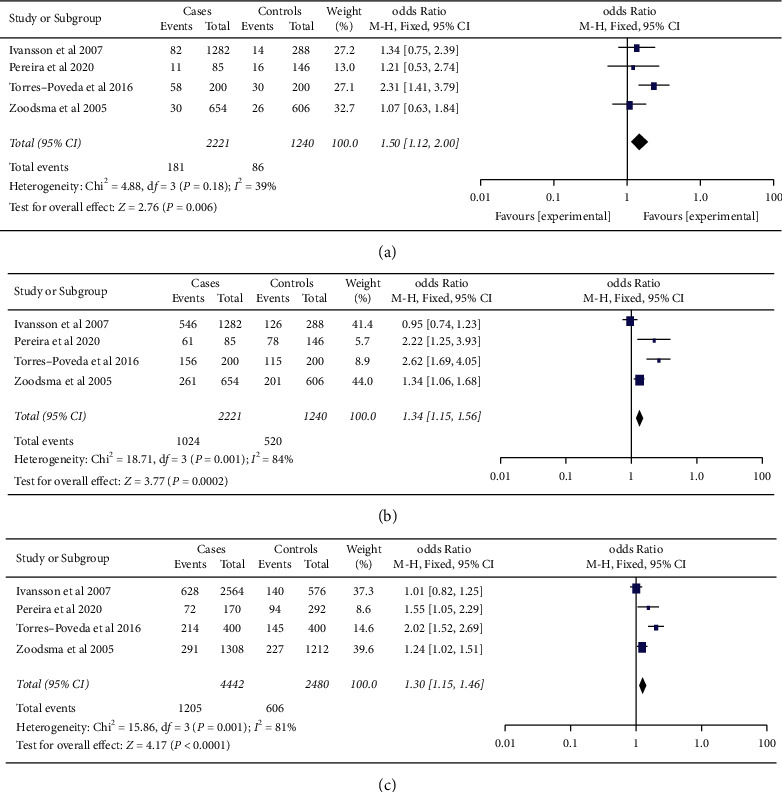
Forest plots of the association between SNP*-592C/A* in the *IL-10* gene and cervical cancer for the (a) recessive model, (b) dominant model, and (c) additive model in the Caucasian population. The pooled OR is represented by a black diamond, the OR in each study is represented by blue squares with square sizes inversely proportionate to the standard error of the OR, and the horizontal lines represent the 95% CI.

**Figure 4:**
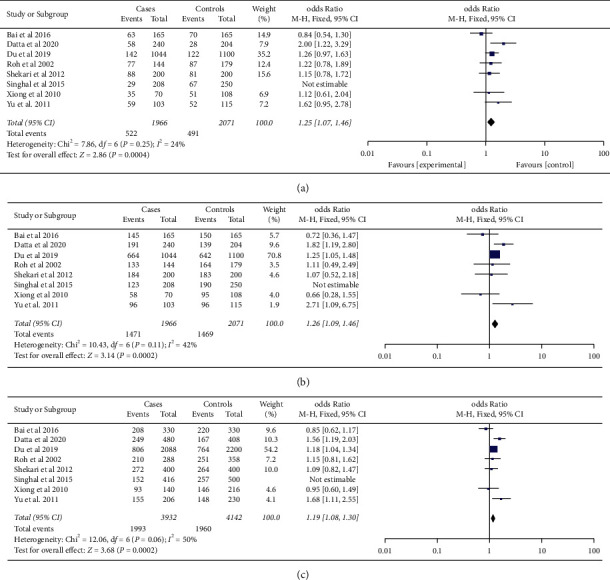
Forest plots of the association between the *-592C/A* polymorphism of the *IL-10* gene and cervical cancer for the (a) recessive model, (b) dominant model, and (c) additive model in the Asian population. The black diamond represents the pooled OR, the blue squares show the OR in each study with square sizes inversely proportional to the standard error of the OR, and the horizontal lines denote the 95% CI.

Based on analysis by race/ethnicity (Table 2), the *-592C/A* polymorphism of the *IL-10* gene was significantly associated with an increased risk of cervical cancer in Caucasians for the recessive model (OR (FEM) = 1.50, 95% CI = 1.12–2.00, *p*=0.006), dominant model (OR (REM) = 1.57, 95% CI = 1.03–2.40, *P*=0.04), and additive model (OR (FEM) = 1.15, 95% CI = 1.15–1.46, *P* < 0.0001) (Figure 3). Furthermore, an association of this polymorphism with cervical cancer was observed in the Asian populations for the recessive (OR (FEM) = 1.25, 95% CI = 1.07–1.46, *P*=0.004), dominant (OR (FEM) = 1.26, 95% CI = 1.09–1.46, *P*=0.002), and additive (OR (FEM) = 1.19, 95% CI = 1.08–1.30, *P*=0.0002) models (Figure 4). After Bonferroni correction adjusts *P* value, a nonsignificant association was found between *IL-10 -592C/A* polymorphism and cancer for the dominant model in Caucasians.

### 3.3. Sensitivity Analysis

The stability of the meta-analysis was maintained by removing studies that significantly changed the overall OR and *P* value after excluding those that deviated from HWE [8]. In this regard, only the article by Singhal et al. has been omitted [19].

### 3.4. Heterogeneity Source

We found heterogeneity for the dominant and additive models with *I*^2^ > 50 and *I*^2^ = 66 percent, respectively, when we excluded studies that deviated from HWE and the ones that significantly affected the cumulative OR value (Figures 2(b) and 2(c)). The genetic models showed no heterogeneity in the race/ethnicity study, except for the dominating pattern in Caucasians (*I*^2^ = 84 percent, *P*=0.0003) (Figure 3(b)).

### 3.5. Publication Bias

**Figure 5:**
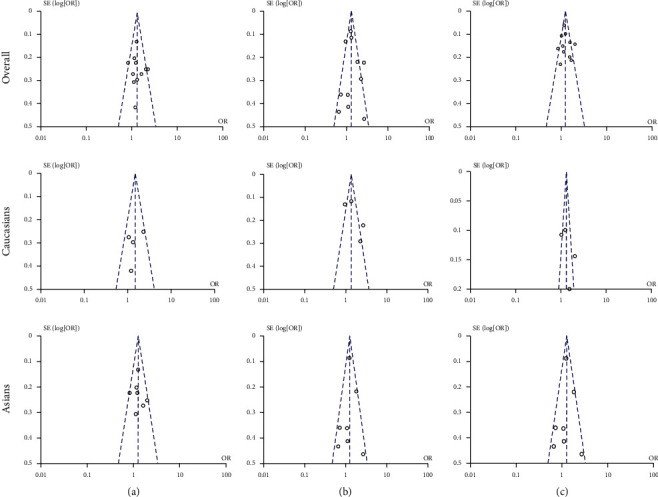
Funnel plots of the (a) recessive model, (b) dominant model, and (c) additive model precision by OR.

The evaluation of publication bias was conducted by performing funnel plots. An absence of publication bias was observed for the recessive, dominant, and additive models after removing studies, not in agreement with the HWE and the study modifying the value of the pooled OR (Figure 5).

## 4. Results of TSA

**Figure 6:**
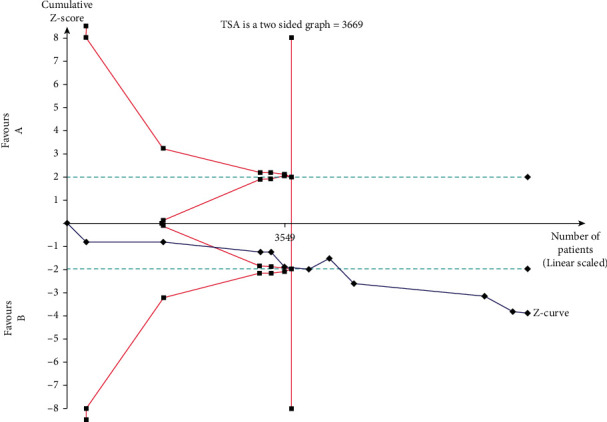
Trial sequential analysis for *IL10-592C/A* polymorphism under the genotype contrast model. The *x*-axis shows the number of participants (cases and controls) of the meta-analysis in each branch. The *y*-axis *z*-score shows the *z*-score. The red line represents the required sample size. The green line represents the conventional test boundary (*P*=0.05).

The TSA's outcome is depicted in Figure 6. The conclusions of the present meta-analysis are strong because the required sample size is 3669 and the cumulative *z*-curve reached the requested sample size by crossing the upper limit of sequential trial monitoring.

## 5. Discussion

HPV infection is one of the leading causes of cervical cancer, yet it is not sufficient to trigger cervical carcinogenesis. Smoking, HIV, fetal exposure to diethylstilbestrol, and oral contraceptives have all been identified as additional risk factors. Although genetic variables have been linked to carcinogenesis, the mechanism by which the IL-10 gene polymorphism causes cervical cancer is unknown. In vitro, IL-10 has been shown to have powerful immunosuppressive and antiinflammatory activities [35–37].

This cytokine is produced by a variety of cell types, such as CD4 T cells and monocytes/macrophages [38, 39]. Macrophages' ability to present antigens to T cells, as well as their ability to provide a costimulatory signal to T cells, is reduced by IL-10. It performs this function by inhibiting the activation of class II MHC-11 molecules and specific accessory molecules on their surfaces, including the B7.1 molecule [40]. By blocking cell-mediated immune responses and inflammatory reactions, IL-10 can promote carcinogenesis by limiting the development of an adequate antitumor response against tumor cells [40, 41].

It is worth noting that, in addition to its inhibitory characteristics, IL-10 stimulates antibody production, as well as the differentiation and proliferation of B lymphocytes, which in turn produce IL-10 [42–44]. Furthermore, the expression of the *IL-10* gene has been confirmed in various tumors, suggesting that IL-10 could play a nonnegligible role in carcinogenesis by allowing the immune system to escape [4]. In promoter regions, functional polymorphisms of the *IL-10* gene such as *-592C/A* can lead to changes in the affinity of transcriptional factors, thereby altering levels of mRNA expression (dose-dependent effect) of inflammatory cytokines associated with the occurrence of cancer [22, 45].

In the present study, including 4187 cases and 3311 controls, we noted that the *−592C/A* polymorphism of the *IL-10* gene was correlated with the overall risk of cervical cancer for the recessive, dominant, and additive models. This finding is in line with a previous meta-analysis that identified a significant association between the *−592C/A* polymorphism and an increased risk of cervical cancer in 2396 cases and 1388 controls [46]. Contrary to our results, Guo et al. reported in a meta-analysis of 3,149 cases and 2,237 controls that the *−592C/A* polymorphism was not globally correlated with cervical cancer for the three genetic models [14]. A recent meta-analysis with 1,393 cases and 1,307 controls also found conflicting results [47]. In Caucasians and Asians, our meta-analysis adjusted for race and ethnicity found an association between the *−592C/A* polymorphism and cervical cancer in all models. Some meta-analyses supported our conclusions in some respects. This was the case in the meta-analysis by Guo et al. on the Caucasian population, which found that only the recessive model was associated with an increased risk of cervical cancer [14], and another meta-analysis from the Asian population, which found that the additive model was associated with the risk of cervical cancer [46]. However, Wang et al. found that the *−592C/A* polymorphism had no effect on the risk of cervical cancer in Caucasian and Asian populations across all genetic models tested [47].

Furthermore, Torres-Poveda et al. and Pereira et al. found a correlation between the *−592C/A* polymorphism and cervical cancer in the Mexican and Brazilian populations for all three models, respectively [17, 21]. Zoodsma et al., Du et al., and Datta et al. reported that the risk of cervical cancer was correlated with the *−592C/A* SNP in the *IL-10* gene for the dominant and additive models in Netherlander, Chinese, and Bangladeshi populations, respectively [6, 7, 30]. These discrepancies between studies can be explained by a few factors, including (1) sample size differences between studies, (2) inclusion of studies with allele frequencies that deviate from the HWE, (3) inclusion of studies that change the pooled OR value, (4) population genetic background, and (5) inclusion of studies based on noncancerous cervical lesions [8, 19, 48].

The present study shows certain limitations as follows: the limited number of case and control studies carried out on the association of SNP*-592C/A* in the *IL-10* gene with the risk of cervical cancer, particularly in the Caucasian and African populations (almost absent), and the sample size. Although the sample size requirement showed statistical power of 90%, a more representative sample size from different populations worldwide could confirm or refute a robust conclusion.

## 6. Conclusions

Based on an analysis of a large sample with precise inclusion criteria, this study reveals that women with *the −529C/A* polymorphism of the *IL-10* gene promoter have a high risk of cervical cancer for genetic models and provides evidence of an association of the *IL-10* gene promoter in the pathogenesis of cervical cancer.

## 7. Disclosure

This manuscript was presented as a preprint in “Association of *Interleukin-10 −592C/A* Polymorphism and Cervical Cancer Risk: A Meta-Analysis” (Preprint) [49]

## Figures and Tables

**Table 1:** Genotypic distribution of the *IL-10-592C/A* polymorphism in eligible studies.

Author/year	Race/ethnicity	Cases				Controls				
		*N*	*CC*	*CA*	*AA*	*N*	*CC*	*CA*	*AA*	HWE
[20]	Asian	165	20	82	63	165	15	80	70	0.24
[6]	Asian	240	49	133	58	204	65	111	28	0.07
[7]	Asian	1044	380	522	142	1100	458	520	122	0.15
[29]	Caucasian	1282	736	464	82	288	162	112	14	0.33
[21]	Caucasian	85	24	50	11	146	68	62	16	0.74
[31]	Asian	144	11	56	77	179	15	77	87	0.72
[32]	Asian	200	16	96	88	200	17	102	81	0.05
[17]	Caucasian	200	44	98	58	200	85	85	30	0.25
[33]	Asian	70	12	23	35	108	13	44	51	0.46
[34]	Asian	103	7	37	59	115	19	44	52	0.07
[30]	Caucasian	654	393	231	30	606	405	175	26	0.20

*N*: number.

**Table 2:** Genetic models and *SNP-592C/A* in the *IL-10* gene in cervical cancer.

Study	*N*	Cases/control	Models	Effect estimate/statistical		Bonferroni		Heterogeneity	
				OR (95% CI)	*P* value	*α*	Sig	*I* ^2^ (%)	*P*′
All studies	11	4187/3311							
			Recessive	1.30 (1.14–1.49)^*∗*^	0.0001	0.016	Yes	28	0.18
			Dominant	1.36 (1.09–1.70)^*∗∗*^	0.006	0.016	Yes	66	<0.05
			Additive	1.25 (1.09–1.44)^*∗∗*^	0.001	0.025	Yes	66	<0.05

Caucasian	4	2221/1240							
			Recessive	1.50 (1.12–2.00)^*∗*^	0.006	0.016	Yes	39	0.18
			Dominant	1.57 (1.03–2.40)^*∗∗*^	0.04	0.016	No	84	<0.05
			Additive	1.15 (1.15–1.46)^*∗∗*^	<0.0001	0.025	Yes	81	<0.05

Asian	7	1966/2071							
			Recessive	1.25 (1.07–1.46)^*∗*^	0.004	0.016	Yes	24	0.25
			Dominant	1.26 (1.09–1.46)^*∗*^	0.002	0.016	Yes	42	0.11
			Additive	1.19 (1.08–1.30)^*∗*^	0.0002	0.035	Yes	50	0.06

*N*: number; *P*: *P* value OR; *P*′: *P* value of heterogeneity; *I*^2^: inconsistency; recessive model: *AA* vs. *CC* + *CA*; dominant model: *AA* + *CA* vs. *CC*; additive model: *A* vs. *C*; ^*∗*^ = fixed-effect model, ^*∗∗*^ = random-effect model; *N* = number; *α* = Bonferroni correction; Sig = Bonferroni significance.

## Data Availability

The supplementary material represents all the data analyzed in this meta-analysis.

## Abbreviations

*AA* vs. *CC* + *CA*: Recessive model
*AA* + *CA* vs. *CC*: Dominant model
*A* vs. *C*: Additive model
CI: Confidence interval
FE: Fixed effect
Fig: Figure
HWE: Hardy–Weinberg equilibrium
*I* ^2^: Inconsistency
IL-10: Interleukin-10
*N*: Number
OR: Odds ratio
RE: Random effect
vs: Versus.

## Supplementary Materials

Supplementary Materials PubMed accession numbers and Google scholar link of all data and references are given for meta-analysis.

## References

[1] Sung H., Ferlay J., Siegel R. L. (2021). Global cancer statistics 2020: GLOBOCAN estimates of incidence and mortality worldwide for 36 cancers in 185 countries. *CA: A Cancer Journal for Clinicians*.

[2] Moore E. E., Wark J. D., Hopper J. L., Erbas B., Garland S. M. (2012). The roles of genetic and environmental factors on risk of cervical cancer: a review of classical twin studies. *Twin Research and Human Genetics*.

[3] Xiao D., Liu D., Wen Z. (2019). Interaction between susceptibility loci in MAVS and TRAF3 genes, and high-risk HPV infection on the risk of cervical precancerous lesions in Chinese population. *Cancer Prevention Research*.

[4] Howell M. W. (2013). Interleukin-10 gene polymorphisms and cancer. *Madame Curie Bioscience Database, Landes Bioscience*.

[5] Machalek D. A., Wark J. D., Tabrizi S. N. (2017). Genetic and environmental factors in invasive cervical cancer: design and methods of a classical twin study. *Twin Research and Human Genetics*.

[6] Datta A., Tuz Zahora F., Abdul Aziz M. (2020). Association study of IL10 gene polymorphisms (rs1800872 and rs1800896) with cervical cancer in the Bangladeshi women. *International Immunopharmacology*.

[7] Du G.-H., Wang J.-K., Richards J. R., Wang J.-J. (2019). Genetic polymorphisms in tumor necrosis factor alpha and interleukin-10 are associated with an increased risk of cervical cancer. *International Immunopharmacology*.

[8] Zidi S., Gazouani E., Stayoussef M. (2015). IL-10 gene promoter and intron polymorphisms as genetic biomarkers of cervical cancer susceptibility among Tunisians. *Cytokine*.

[9] Saraiva M., Christensen J. R., Veldhoen M., Murphy T. L., Murphy K. M., O’Garra A. (2009). Interleukin-10 production by Th1 cells requires interleukin-12-induced STAT4 transcription factor and ERK MAP kinase activation by high antigen dose. *Immunity*.

[10] Stumhofer J. S., Silver J. S., Laurence A. (2007). Interleukins 27 and 6 induce STAT3-mediated T cell production of interleukin 10. *Nature Immunology*.

[11] Eskdale J., Kube D., Tesch H., Gallagher G. (1997). Mapping of the human IL10 gene and further characterization of the 5’ flanking sequence. *Immunogenetics*.

[12] Trifunović J., Miller L., Debeljak Ž., Horvat V. (2015). Pathologic patterns of interleukin 10 expression—a review. *Biochemical Medicine*.

[13] Abbas M., Mason T., Ibad A. (2020). Genetic polymorphisms in IL-10 promoter are associated with smoking and prostate cancer risk in african Americans. *Anticancer Research*.

[14] Guo C., Wen L., Song J.-K. (2018). Significant association between interleukin-10 gene polymorphisms and cervical cancer risk: a meta-analysis. *Oncotarget*.

[15] Li M., Yue C., Zuo X. (2020). The effect of interleukin 10 polymorphisms on breast cancer susceptibility in Han women in Shaanxi Province. *PLoS One*.

[16] Nagano T., Kunisada M., Yu X., Masaki T., Nishigori C. (2007). Involvement of interleukin-10 promoter polymorphisms in nonmelanoma skin cancers-a case study in non-Caucasian skin cancer patients. *Photochemistry and Photobiology*.

[17] Torres-Poveda K., Burguete-García A. I., Bahena-Román M. (2016). Risk allelic load in Th2 and Th3 cytokines genes as biomarker of susceptibility to HPV-16 positive cervical cancer: a case control study. *BMC Cancer*.

[18] Wang X., Yang F., Xu G., Zhong S. (2018). The roles of IL-6, IL-8 and IL-10 gene polymorphisms in gastric cancer: a meta-analysis. *Cytokine*.

[19] Singhal P., Kumar A., Bharadwaj S., Hussain S., Bharadwaj M. (2015). Association of IL-10 GTC haplotype with serum level and HPV infection in the development of cervical carcinoma. *Tumor Biology*.

[20] Bai C. Y., Shi X. Y., He J., Xue J., Feng Y. (2016). Association between IL-10 genetic variations and cervical cancer susceptibility in a Chinese population. *Genetics and Molecular Research*.

[21] Pereira A. P. L., Trugilo K. P., Okuyama N. C. M. (2020). IL-10 c.-592C>A (rs1800872) polymorphism is associated with cervical cancer. *Journal of Cancer Research and Clinical Oncology*.

[22] de Oliveira J. G., Rossi A. F. T., Nizato D. M. (2015). Influence of functional polymorphisms in TNF-*α*, IL-8, and IL-10 cytokine genes on mRNA expression levels and risk of gastric cancer. *Tumor Biology*.

[23] Hiroki C. H., Amarante M. K., Petenuci D. L. (2015). IL-10 gene polymorphism and influence of chemotherapy on cytokine plasma levels in childhood acute lymphoblastic leukemia patients: IL-10 polymorphism and plasma levels in leukemia patients. *Blood Cells, Molecules, and Diseases*.

[24] Niu Y.-M., Du X.-Y., Cai H.-X. (2015). Increased risks between Interleukin-10 gene polymorphisms and haplotype and head and neck cancer: a meta-analysis. *Scientific Reports*.

[25] Cumpston M., Li T., Page M. J. (2019). Updated guidance for trusted systematic reviews. *Cochrane Database of Systematic Reviews*.

[26] DerSimonian R., Laird N. (2015). Meta-analysis in clinical trials revisited. *Contemporary Clinical Trials*.

[27] Egger M., Smith G. D., Schneider M., Minder C. (1997). Bias in meta-analysis detected by a simple, graphical test. *British Medical Journal*.

[28] Meng J., Wang S., Zhang M., Fan S., Zhang L., Liang C. (2018). TP73 G4C14-A4T14 polymorphism and cancer susceptibility: evidence from 36 case–control studies. *Bioscience Reports*.

[29] Ivansson E. L., Gustavsson I. M., Magnusson J. J. (2007). Variants of chemokine receptor 2 and interleukin 4 receptor, but not interleukin 10 or Fas ligand, increase risk of cervical cancer. *International Journal of Cancer*.

[30] Zoodsma M., Nolte I. M., Schipper M. (2005). Interleukin-10 and Fas polymorphisms and susceptibility for (pre)neoplastic cervical disease. *International Journal of Gynecological Cancer*.

[31] Roh J. W., Kim M. H., Seo S. S. (2002). Interleukin-10 promoter polymorphisms and cervical cancer risk in Korean women. *Cancer Letters*.

[32] Shekari M., Kordi-Tamandani D. M., MalekZadeh K., Sobti R. C., Karimi S., Suri V. (2012). Effect of anti-inflammatory (IL-4, IL-10) cytokine genes in relation to risk of cervical carcinoma. *American Journal of Clinical Oncology*.

[33] Xiong X.-d., Lu S.-x., Zeng L.-q. (2010). Relationship between IL-10-592A>C promoter polymorphism and the susceptibility to cervical cancer. *Chinese Journal of Birth Health & Heredity*.

[34] Yu X.-m., Ma D., Wu S.-q. (2011). Relationship between polymorphisms of IL-10 gene and cervical cancer. *Chinese Journal of Nosocomiology*.

[35] de Vries J. E. (1995). Immunosuppressive and anti-inflammatory properties of interleukin 10. *Annals of Medicine*.

[36] Hervás-Salcedo R., Fernández-García M., Hernando-Rodríguez M. (2021). Enhanced anti-inflammatory effects of mesenchymal stromal cells mediated by the transient ectopic expression of CXCR4 and IL10. *Stem Cell Research & Therapy*.

[37] Katayama T., Hayashi Y., Nagahira K., Konishi K., Yamaichi K., Oikawa S. (2003). Imidocarb, a potent anti-protozoan drug, up-regulates interleukin-10 production by murine macrophages. *Biochemical and Biophysical Research Communications*.

[38] Gabryšová L., Howes A., Saraiva M., O’Garra A. (2014). The regulation of IL-10 expression. *Current Topics in Microbiology and Immunology*.

[39] Rutz S., Ouyang W. (2016). Regulation of interleukin-10 expression. *Advances in Experimental Medicine and Biology*.

[40] Costa Brandão Berti F., Brajão de Oliveira K., Berti F. C. B., de Oliveira K. B. (2018). IL-10 in cancer: just a classical immunosuppressive factor or also an immunostimulating one?. *AIMS Allergy and Immunology*.

[41] Lin W.-W., Karin M. (2007). A cytokine-mediated link between innate immunity, inflammation, and cancer. *Journal of Clinical Investigation*.

[42] Couper K. N., Blount D. G., Riley E. M. (2008). IL-10: the master regulator of immunity to infection. *The Journal of Immunology*.

[43] Rousset F., Garcia E., Defrance T. (1992). Interleukin 10 is a potent growth and differentiation factor for activated human B lymphocytes. *Proceedings of the National Academy of Sciences of the U S A*.

[44] Sabat R., Grütz G., Warszawska K. (2010). Biology of interleukin-10. *Cytokine & Growth Factor Reviews*.

[45] Fan S., Meng J., Zhang L., Zhang X., Liang C. (2019). CAV1 polymorphisms rs1049334, rs1049337, rs7804372 might be the potential risk in tumorigenicity of urinary cancer: a systematic review and meta-analysis. *Pathology, Research & Practice*.

[46] Ni J., Ye Y., Teng F., Wu Q. (2013). Interleukin 10 polymorphisms and cervical cancer risk: a meta-analysis. *International Journal of Gynecological Cancer*.

[47] Wang K., Jiao Z., Chen H. (2021). The association between rs1800872 polymorphism in interleukin-10 and risk of cervical cancer: a meta-analysis. *Medicine (Baltimore)*.

[48] Duvlis S., Dabeski D., Noveski P., Ivkovski L., Plaseska-Karanfilska D. (2020). Association of IL-10 (rs1800872) and IL-4R (rs1805010) polymorphisms with cervical intraepithelial lesions and cervical carcinomas. *Journal of B.U.ON*.

[49] Diakite B., Kassogue Y., Maiga M. (2022). Association of interleukin-10 -592C/A polymorphism and cervical cancer risk: a meta- analysis. https://www.researchsquare.com.

