# Supplementary material for: Association of the Interleukin-10-592C/A Polymorphism and Cervical Cancer Risk: A Meta-Analysis
Source: Genet Res (Camb). 2022 Jul 12;2022:2319161. doi: 10.1155/2022/2319161 (PMC9296312; doi:10.1155/2022/2319161)
Supplement: Supplementary Materials — PubMed accession numbers and Google scholar link of all data and references are given for meta-analysis. [file 2319161.f1.doc]

**Supplementary material.** PubMed accession numbers and Google scholar link of all data and references retaining for meta-analysis.

| **Author** | PubMed accession/  Google Scholar |
| --- | --- |
| Bai et al., 2016 | PMID: **27525910** |
| Datta et al., 2020 | PMID: 33069925 |
| Du et al., 2019 | PMID: 30453149 |
| Ivansson et al., 2007 | PMID: **17688234** |
| Pereira et al., 2020 | PMID: **32447484** |
| Roh et al., 2002 | PMID: **12104048** |
| Shekari et al., 2012 | PMID: **22157213** |
| Torres-Poveda et al., 2016 | PMID: **27220278** |
| Xiong et al., 2010 | Google scholar |
| Yu et al., 2011 | Google scholar |
| Zoodsma et al., 2005 | PMID: **16343245** |

Supplementary Material represents the data supporting the conclusions of this meta-analysis. These data come from scientific databases available in the public domain, *Pubmed* and *Google sholar.*
